# Mortality of civilian patients with suspected traumatic haemorrhage receiving pre-hospital transfusion of packed red blood cells compared to pre-hospital crystalloid

**DOI:** 10.1186/s13049-018-0567-1

**Published:** 2018-11-20

**Authors:** J. E. Griggs, J. Jeyanathan, M. Joy, M. Q. Russell, N. Durge, D. Bootland, S. Dunn, E. D. Sausmarez, G. Wareham, A. Weaver, R. M. Lyon, on behalf of Kent, Surrey & Sussex Air Ambulance Trust

**Affiliations:** 1 Kent, Surrey & Sussex Air Ambulance Trust, Redhill Aerodrome, Redhill, RH1 5YP UK; 2 Academic Department of Military Anaesthesia and Critical Care, London, UK; 3 University of Surrey, Guildford, GU2 7XH UK; 4 Royal London Hospital, Whitechapel Road, Whitechapel, London, E1 1BB UK; 5 Royal Sussex County Hospital, Eastern Road, Brighton, BN2 5BE UK

**Keywords:** Transfusion, Packed Red Blood Cells, Crystalloid, Mortality, Traumatic Haemorrhage, Helicopter Emergency Medical Services

## Abstract

**Background:**

Major haemorrhage is a leading cause of mortality following major trauma. Increasingly, Helicopter Emergency Medical Services (HEMS) in the United Kingdom provide pre-hospital transfusion with blood products, although the evidence to support this is equivocal. This study compares mortality for patients with suspected traumatic haemorrhage transfused with pre-hospital packed red blood cells (PRBC) compared to crystalloid.

**Methods:**

A single centre retrospective observational cohort study between 1 January 2010 and 1 February 2015. Patients triggering a pre-hospital Code Red activation were eligible. The primary outcome measure was all-cause mortality at 6 hours (h) and 28 days (d), including a sub-analysis of patients receiving a major and massive transfusion. Multivariable regression models predicted mortality. Multiple Imputation was employed, and logistic regression models were constructed for all imputed datasets.

**Results:**

The crystalloid (*n =* 103) and PRBC (*n =* 92) group were comparable for demographics, Injury Severity Score (*p =* 0.67) and mechanism of injury (*p* = 0.73). Observed 6 h mortality was smaller in the PRBC group (*n* = 10, 10%) compared to crystalloid group (*n* = 19, 18%). Adjusted OR was not statistically significant (OR 0.48, CI 0.19–1.19, *p* = 0.11). Observed mortality at 28 days was smaller in the PRBC group (*n* = 21, 26%) compared to crystalloid group (*n* = 31, 40%), *p* = 0.09. Adjusted OR was not statistically significant (OR 0.66, CI 0.32–1.35, *p* = 0.26). A statistically significant greater proportion of the crystalloid group required a major transfusion (*n* = 62, 60%) compared to the PRBC group (*n* = 41, 40%), *p* = 0.02. For patients requiring a massive transfusion observed mortality was smaller in the PRBC group at 28 days (*p =* 0.07).

**Conclusion:**

In a single centre UK HEMS study, in patients with suspected traumatic haemorrhage who received a PRBC transfusion there was an observed, but non-significant, reduction in mortality at 6 h and 28 days, also reflected in a massive transfusion subgroup. Patients receiving pre-hospital PRBC were significantly less likely to require an in-hospital major transfusion. Further adequately powered multi-centre prospective research is required to establish the optimum strategy for pre-hospital volume replacement in patients with traumatic haemorrhage.

## Background

Traumatic haemorrhage is the leading cause of preventable death in major trauma patients [1, 2]. Approximately half of all patient deaths in the first 24-h are due to haemorrhage [3]. Survival from major traumatic haemorrhage is poor. Trauma patients who require substantial transfusion have a mortality greater than 30 % [4]. National epidemiology studies in England and Wales estimate the annual incidence of major traumatic haemorrhage as 4700 patients, with 1300 patients proceeding to massive haemorrhage [5]. Traumatic haemorrhage is further compounded by coagulopathy [6, 7]. Targeted resuscitation of patients in a post-traumatic coagulopathic state is critical to improving patient outcome [8, 9].

Historically, the hypotensive trauma patient with suspected traumatic haemorrhage was administered crystalloid [10, 11]; however, not without significant adverse effects [6, 12, 13]. Trauma Induced Coagulopathy (TIC) can be sub-divided to endogenous acute traumatic coagulopathy (ATC) and subsequent dilutional coagulopathy [14]. Crystalloid infusion can worsen dilutional coagulopathy [15], endothelial damage and tissue oedema [7], further compounding multiple organ dysfunction and trauma–related bleeding [16, 17]. In-hospital literature highlights worse outcomes for patients receiving greater volumes of crystalloid [18]; negating its administration [11].

Increasingly, Helicopter Emergency Medical Services (HEMS) in the United Kingdom (UK) provide pre-hospital blood product transfusion. Administration of packed red blood cells (PRBC) has emulated from military [19] to civilian practice [20, 21]. The transfusion of PRBC transfusion has become the fluid resuscitation method of choice, and more recently, the addition of freeze dried plasma (FDP) or fresh frozen plasma (FFP) [22]. Early transfusion therapy is postulated to bridge the gap to damage control resuscitation [21, 23]. Literature reports that a delay in transfusion of PRBC (> 10 min) was associated with increased odds of death for transfused patients; supporting expedient transfusion capability [24].

Heterogeneity exists in the UK, with approximately 50% of HEMS services administering blood products versus crystalloid (0.9% sodium chloride) [25]. Equivocal literature, and the combined logistical complexities, storage and clinician availability to provide pre-hospital transfusion of PRBC, has led to widespread heterogeneity across UK HEMS practice. Naumann et al. (25) assert that evidence-based justification of pre-hospital PRBC would see approximately 800 eligible transfusions per year. Despite blood product transfusion being noted as a clinically logical step, PRBC transfusion itself is not without clinical complications. Transfusion reactions, independent association to acute respiratory distress syndrome, incremental infectious complications [26] and multiple organ dysfunction is noted [7, 27].

Clinical literature for the use of pre-hospital PRBC is ambiguous [2, 16]. Systematic review identifies no published prospective, blinded or randomised studies comparing pre-hospital crystalloid and PRBC resuscitation [2, 28]. Furthermore, studies have focused on small patient cohorts highlighting only the feasibility and safety of pre-hospital PRBC transfusion [6, 29–32].

Pre-hospital studies include disparate patient cohorts with confounding interventions and contrasting outcomes [6, 33, 34], which limits meta-analysis [28, 35]. Subsequently, substantial heterogeneity limits long term mortality statistical analysis, this is further hampered by loss to follow up ranging from 18% [36] to 67% [37], respectively. A prospective randomised controlled trial (RCT), Resuscitation with Pre-hospital Blood Products [38] will compare crystalloid (0.9% sodium chloride) against PRBC and FDP, with the primary outcome measures of lactate clearance and all-cause mortality.

To date, clinical literature regarding transfusion of PRBC in civilian patients is equivocal. The objective of this retrospective observational study is to ascertain any association between mortality and patients transfused with pre-hospital PRBC compared to crystalloid (0.9% sodium chloride) in civilian patients with suspected traumatic haemorrhage.

## Methods

### Study design and pre-hospital care system

This is a single centre, retrospective observational cohort study of patients triggering a pre-hospital ‘Code Red’ activation. The study was registered with the University of Surrey and met UK National Institute of Healthcare Research (NIHR) criteria as a service evaluation. The study applied Strengthening the Reporting of Observational Studies in Epidemiology (STROBE) Guidelines [39].

The Kent, Surrey and Sussex Air Ambulance Trust (KSSAAT) provides a HEMS service in southeast England, UK. The HEMS clinicians (Physician and Paramedic) deploy by aircraft or response vehicle. Operational teams cover the region over 24 h, with a second team providing operational cover over a further 18 h day. Enhanced medical care is provided to approximately 2000 patients per year in a predominantly rural and static population of 4.5 million, with a transient population of 10 million. Patients were conveyed to one of five Major Trauma Centres (MTC).

### Code red standard operating procedure

In this service, where there is a clinical suspicion of major haemorrhage and signs of haemodynamic compromise ‘Code Red’ is declared. Code Red is informed by pre-hospital clinical assessment and declared at the discretion of the attending HEMS clinicians. A Code Red activation comprised of the following parameters during the study period.

In hypotensive patients with suspected traumatic haemorrhage (systolic blood pressure (SBP) < 80 mmHg or absence of a radial pulse) the concept of ‘permissive hypotension’ is targeted, i.e. SBP of ≥80 mmHg, or the return of a radial pulse. In patients with polytrauma and suspected traumatic brain injury an SBP of ≥100 mmHg is targeted, and in patients with penetrating torso trauma, a carotid pulse. Alternative causes of hypotension are excluded, such as tension pneumothorax.

From January 2013, following a robust programme of work at KSSAAT, and pragmatic view of available in-hospital and military literature, a decision was made to introduce pre-hospital PRBC transfusion as a clinical logical step in the management of patients with suspected traumatic haemorrhage. A Code Red activation ensured PRBC transfusion through a Belmont Buddy Lite™ fluid warmer (Belmont Instrument Corporation, M. A, USA) and the administration of tranexamic acid. The activation enables a titrated transfusion of up to four units of O Rhesus negative PRBC from the CRĒDO CUBE™ (Series 4, 2 l Insulation 15, VIP Golden Hour). Subsequently, a ‘pre-alert’ call to the receiving hospital triggers a predefined in-hospital major haemorrhage protocol; ensuring blood and clotting factors are immediately available [30, 32]. Adherence and compliance to the Blood Safety and Quality Regulations (2017) [40] and Medicines and Healthcare Regulatory Agency was ensured [41].

### Data collection

Between 1 January 2010 and 31 January 2013, Code Red patients were administered crystalloid (*crystalloid group,* sodium chloride 0.9%, in 250 ml boluses titrated to effect). Between 1 February 2013 and 1 February 2015 Code Red patients were transfused with PRBC (*PRBC group*, transfused up to a maximum of 4 units O Rhesus negative PRBC). Paper clinical records were interrogated from January 2010 until July 2013, subsequently a bespoke electronic patient record system was introduced (HEMSBase, Medic One Systems Limited, UK) [42]. HEMSBase was interrogated from July 2013 to February 2015. In February 2015, freeze dried plasma (FDP) was introduced into the service, at this point data collection for eligible patients was ceased.

Patient demographics and clinical data were collected for eligible patients. The SBP (mmHg) reflects the first HEMS recorded value. The recorded volume (mL) of crystalloid is that administered by HEMS clinicians only, and not pre-existing administration by the attending ambulance clinicians. Incident descriptors (mechanism of injury (MOI)), 999 time to HEMS on scene time, and Injury Severity Score (ISS) were reported. Primary outcome of all-cause mortality at 6 h (h) and 28 days (d) was recorded. A sub-analysis of patients receiving in-hospital major transfusion (≥4 units PRBC in 24 h) and massive transfusion (≥10 units PRBC in 24 h), not including pre-hospital PRBC, was reported [15].

Pre-hospital and in-hospital data were reviewed retrospectively. In-hospital data was collected from the Trauma Audit and Research Network (TARN) database. Pre-existing data sharing agreements enabled interrogation of hospital-specific computer-based records for supplementary data. Data was abstracted by the first reviewer (JG); inaccuracies and discrepancies were resolved by a second reviewer (JJ).

### Inclusion criteria

Inclusion criteria comprised: 1) blunt and/or penetrating traumatic injury with suspected traumatic haemorrhage, 2) pre-hospital Code Red declaration with transfusion of crystalloid and/or PRBC, 3) patient conveyed to an MTC, 4) traumatic cardiac arrests (TCAs) where return of spontaneous circulation (ROSC) was gained, declared Code Red and conveyed to an MTC.

Exclusion criteria comprised: 1) paediatrics (< 16 years), 2) patients declared Code Red with a suspected medical aetiology, 2) TCA; where patients were pronounced life extinct, 3) patients transferred to non-MTCs, 4) inter-hospital and/or secondary transfers.

### Primary outcome measure

The co-primary outcome measures were in-hospital all-cause mortality at 6 h and 28 d. In order to identify patients with ‘true’ ongoing haemorrhage a sub-analysis of all-cause mortality for patients receiving a massive transfusion or major transfusion over the first 24 h period was reported.

### Statistical analysis

Descriptive statistics are reported; counts, percentages and ages are presented for categorical data. Continuous data is reported by mean and median (IQR). Chi squared tests were performed for categorical variables. Kruskal-Wallis tests compared continuous variables between the crystalloid and PRBC group.

Risk adjustment was performed by creating a multivariate logistic regression model to predict both mortalities, utilising the covariates age, SBP, ISS, MOI. Adjusted Odds Ratios (OR) and Confidence Intervals (CI) are reported.

Statistical analysis was performed using R, version 3.4.0 [43]. Multiple imputation (MI) was employed to limit the effect of missing data in several covariates using the MICE package in R. Predictive mean matching was used, and ten data sets were imputed. Kernel density plots revealed a satisfactory imputation for ISS, MOI, massive transfusion, major transfusion and 28 d mortality.

Logistic regression models were constructed for all imputed datasets, and coefficients estimates pooled according to Rubin’s rules [44]. Statistical significance was assumed as *p* < 0.05.

### Ethical approval

This study met National Institute of Health Research (UK) criteria for Service Evaluation. Internal approval by KSSAAT Research Audit and Development Committee was gained. Formal ethical approval was not required. Patient identifiable data was anonymised and stored on electronic devices with technical encryption (Data Protection Act, 1998).

## Results

**Fig. 1:**
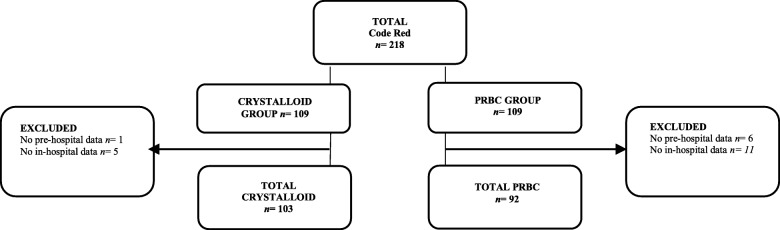
Study population meeting inclusion criteria

During the study period, 218 patients met the inclusion criteria (Fig. 1). The crystalloid group comprised 109 patients, with 6 patients excluded for missing data (*n* = 103). The PRBC group comprised 109 patients, of which 17 patients were excluded for missing data (*n =* 92).

The reasons for exclusion comprised: 1) incomplete pre-hospital data, from patient clinical records, and 2) incomplete in-hospital data, from TARN and/or in-hospital electronic records. During the study period there were no immediate transfusion complications, and 100% traceability of pre-hospital PRBC was achieved.

Missing data in the crystalloid group was noted for 28 d mortality (26%); major transfusion (5%) and massive transfusion (5%). Missing data in the PRBC group is noted for 28 d mortality (15%); major transfusion (3%) and massive transfusion (3%). MI was therefore employed.

### Demographics and incident descriptors

Patient demographics are reported (Table 1). Both groups were predominantly male (*p* = 1.0) and similar in age, mean 44 years (*p* = 0.50). Patient characteristics were comparable for SBP (*p* = 0.56) and ISS, 31 and 32, respectively (*p =* 0.67). Incident descriptors report no difference between the MOI in each group (*p* = 0.73).

**Table 1:** Categorical variables and covariates for the crystalloid and PRBC group; SBP, Systolic Blood Pressure; ISS, Injury Severity Score; MOI, Mechanism of Injury; RTC, Road Traffic Collision; IQR, Interquartile Range; N/A, Not Available

	Crystalloid Group	PRBC Group	*P* value
	*n* = 103	*n* = 92	
Gender			
Female (*n*, %)	26 (25)	24 (26)	
Male (*n, %*)	77 (74)	68 (73)	1.00
Age (mean, SD)	45 (20)	43 (20)	0.50
SBP (mean, SD)	88.21 (25)	90.65 (32)	0.56
ISS (mean, SD)	31.37 (14)	32.26 (12)	0.67
Median 999 time to HEMS on scene time (minutes, IQR)	30 (IQR 23.25–41.75)	35 (IQR 24–51.5)	
MOI (*n*, %)			
RTC Driver	17 (16)	18 (19)	0.73
RTC Passenger	10 (9)	11 (11)	
RTC Pedestrian	8 (7)	18 (19)	
RTC Motorcyclist	22 (21)	13 (14)	
Fall	10 (9)	9 (9)	
Penetrating Injury	2 (1)	5 (5)	
Pedal Cyclist	6 (5)	5 (5)	
Other	9 (8)	7 (7)	
N/A	19 (18)	6 (6)	
Mortality			
6 h mortality			
No (*n*, %)	84 (81)	82 (89)	0.2
Yes (*n*, %)	19 (18)	10 (10)	
28 d mortality			
No (*n*, %)	45 (59)	57 (73)	0.09
Yes (*n*, %)	31 (40)	21 (26)	

In the crystalloid group, an average of 737 mL (IQR 250–1000 mL) of crystalloid was administered by HEMS, compared to 52 mL crystalloid and a median 2.3 PRBC units (IQR 1–3) in the PRBC group. The median PRBC received over the first in-hospital 24 h is documented for the crystalloid group as 4.5 units (IQR 2–9) and for the PRBC group as 3 units (IQR 1–8).

### Primary outcome measure

Unadjusted analysis for observed 6 h mortality was less in the PRBC group (*n* = 10, 10%) versus the crystalloid group (*n* = 19, 18%) but not significantly so, *p* = 0.2. Similarly, for unadjusted 28 d mortality, there was an observed reduction in mortality in the PRBC group (*n* = 21, 26%) versus the crystalloid group (*n* = 31, 40%), *p* = 0.09. However, adjusted odds ratios (OR), after MI for both 6 h and 28 d mortality show no statistically significant association (Table 2).

**Table 2:** Odds ratios for 6 h and 28 d mortality (after multiple imputation adjusted for age, ISS, SBP, MOI)

Mortality	OR	Lower 95% CI	Upper 95% CI	*P* value
6 h	0.48	0.19	1.19	0.11
28 d	0.66	0.32	1.35	0.26

### Massive and major transfusion sub-analysis

Observed frequencies report a statistically significant, greater proportion, of the crystalloid group requiring a major transfusion (*n* = 62, 60% versus, *n* = 41, 40%), *p* = 0.02. There was no statistical difference in the proportion of the crystalloid group requiring a massive transfusion (*n* = 22, 22%) compared to the PRBC group (*n* = 14, 15%), *p* = 0.31.

Adjusted odds ratios, after MI, show no statistically significant association for major transfusion in 6 h mortality (*p* = 0.11) and 28 d mortality (*p* = 0.22). For massive transfusion, there is no statistically significant association for massive transfusion in 6 h mortality (*p* = 0.11). For massive transfusion, there is a non-statistically significant association for transfusion of PRBC and 28 d mortality (*p* = 0.07) (Table 3).

**Table 3:** Odds ratios for 6 h and 28 d mortality in the massive transfusion and major transfusion (after multiple imputation adjusted for age, ISS, SBP, MOI)

	OR	Lower 95% CI	Upper 95% CI	*P*- value
Major Transfusion				
6 h mortality	0.35	0.10	1.27	0.11
28 d mortality	0.55	0.21	1.43	0.22
Massive Transfusion				
6 h mortality	0.04	0.00	2.10	0.11
28 d mortality	0.02	0.00	1.48	0.07

## Discussion

Observed mortality rates are less in the PRBC group at 6 h and 28 days, but not significantly so. Equally, mortality of patients in the major and massive transfusion sub-analysis shows an observed reduction, but not significantly so. Patients receiving pre-hospital PRBC were significantly less likely to receive a major transfusion. To our knowledge this is the first UK HEMS paper to report on patient outcomes following the introduction of pre-hospital PRBC transfusion.

Patient demographics in our study were consistent with published literature. A large proportion of the patients were male [29, 31, 45] with a mean age of 44 years [29, 31, 45]. The ISS of 31 (crystalloid group) and 32 (PRBC group) is close to the mean ISS of 27.5 reported in a systematic review [2] and other studies on pre-hospital fluid resuscitation [32, 45], confirming that substantial anatomical injuries are present in patients with traumatic haemorrhage [2].

Incident descriptors in this study are consistent with the published literature, with a high proportion of blunt traumatic injuries [31]. Median pre-hospital PRBC transfusion comprised 2 units; similar to other UK data [45], consistent with HEMS clinicians focusing on a short scene time to deliver a package of care derived from damage control resuscitation techniques. Overall mortality is approaching 40% for the crystalloid group, consistent with published literature [2], and 27% for the PRBC group.

There was an observed reduction in the crude frequency for mortality at 6 h in the PRBC group, however, adjusted OR after MI was not statistically significant (*p* = 0.11**)**. Other studies have demonstrated no statistically significant difference in 6 h mortality [8]. Early deaths are likely due to exsanguination; requiring future innovation early in the critical window [14]. In the absence of other pre-hospital homeostatic interventions, transfusing large volumes of blood product pre-hospital [45] may ‘bridge the gap’ to definite haemorrhagic control. Equally, in future studies, blood product transfusion in addition to such techniques may well provide survival benefit [45].

There was an observed reduction in the crude frequency for mortality at 28 d in the PRBC group, however, adjusted OR after MI was not statistically significant (*p* = 0.26). One systematic review of 27 observational studies suggests no overall statistically significant survival benefit; however, the review evidences improved survival at 24 h [38]. Other small single centre pilot studies found no difference in 24 h (OR 0.57, *p =* 0.12) or 30 d mortality (OR 0.71, *p =* 0.44), despite improved early outcomes. Group characteristics and mode of transport make group comparability difficult. Other studies have revealed no survival benefit [6, 46]. We hypothesise that the number of patients in our study resulted in insufficient power to detect a true difference. As reported by Smith et al. (28), review of ‘grey’ low quality evidence with small patient populations may hide any survival benefit.

Interestingly we note a reduction in 6 h mortality in the major transfusion and massive transfusion subgroup (*p =* 0.11). In the massive transfusion subgroup 28 d mortality shows mild evidence for improved survival (*p =* 0.07). Arguably at 28 days, death is not due to exsanguination alone; instead coagulopathy, inflammation, immunosuppression and MODS are intrinsically linked [14]. It is plausible that early PRBC transfusion in the immediate resuscitation phase mitigates elements of the post-traumatic coagulopathy by avoiding the haemodilution of erythrocytes with oxygen carrying capability noted in aggressive crystalloid resuscitation [14].

In recent literature the mortality rate for patients with a major haemorrhage approached 50%, this evidence has a similar proportion of patients requiring a massive transfusion to those in our study [14]. It was discussed that during the critical window, blood component therapy was below recommended thresholds, thus, haemostatic competence was not maintained. This may also be one explanation for our observed values.

Brown’s multicentre prospective cohort study (2015) found an independent association between PRBC and the reduction in risk of mortality in a civilian population. Of 1415 patients, 50 received PRBC transfusion and were matched to a cohort of 113 subjects [6]. Propensity score matching documented 98% reduction in odds of 24 h mortality (*p =* 0.04), and 88% reduction in the risk of 30 d mortality (*p* = 0.01). However, raw mortality was not reported, nor were variables used in multivariate regression analysis. In addition, overall mortality for patients requiring a pre-hospital transfusion is reported as 4%, inconsistent with, and considerably lower than, our study and other literature [2]. Notably, half of the transfused patients were inter-hospital transfusions introducing survival bias and reducing external validity in comparison to a primary HEMS cohort of patients.

Conversely, the Pre-hospital Resuscitation on Helicopter Study (PROHS) group reported a multicentre prospective observational study of pre-hospital transfusion in civilian patients [35]. Propensity score matching of 109 patients identified no significant difference between pre-hospital transfusions in a PRBC and plasma group, compared to crystalloid for mortality at 3 h, 24 h and 30 d [35]. Of these patients, 24% received plasma only and 7% PRBC only. Coupled with unexpected differences in SBP, GCS and ISS, only 10% of patients could be matched leading to inconclusive results.

Early haemorrhagic death comprises a notable proportion of patients who may benefit from early transfusion; therefore, including these deaths is critical [47]. By adopting a conditional 30-day survival analysis among 24 h survivors, studies have introduced a survival bias by excluding early haemorrhagic deaths [47, 48]. Rehn et al. (2018) report increased survival to hospital in a before and after study of pre-hospital PRBC transfusion [45]. The ‘delayed death’ concept would result in a larger proportion of patients surviving to hospital, but then going on to die shortly after, resulting in the observed mortality at 6 h shown in our study. This concept provides impetus to advancing in-hospital strategies to improve survival [45].

There was a significant difference between the frequency of patients receiving a major transfusion in the crystalloid (63%) versus PRBC group (46%), *p* = 0.02. This is consistent with previous work [45]. Critically, this likely reflects advancing in-hospital major haemorrhage protocols. The authors are aware that stratification on post-treatment surrogates for injury severity (massive transfusion, ISS) introduces bias [47]. For example, even an international multi-centre retrospective analysis of over 3000 patients could not define a threshold at which massive transfusion equals poorer outcomes [5]. However, in the absence of other measures, massive and major transfusion was used here to retrospectively identify haemorrhagic patients [49]. Arguably, there is no universal approach to massive transfusion; hence, emerging evidence for the clinical application of TEG and ROTEM to detect ATC [49].

### Study limitations

Methodological limitations are inherent within an observational retrospective study. The results of any post hoc design is to be appraised with caution, due to inherent confounding and uncontrolled bias. Although there were no pre-hospital system alterations during the study period other than the resuscitation fluid, there is a natural assumption of unaccounted, uncontrolled change and general improvement to resuscitation care and clinical practice. By excluding the PRBC introduction and implementation phase, variability in clinical practice could have been limited during this study period [45].

The authors are cognisant that this paper crosses a study period where, by virtue of time, there were considerable in-hospital advances. Major Trauma Networks, including MTCs were introduced across London during 2010 and extended throughout in the UK in 2012 which would have enabled wide clinical benefit for patients requiring time critical intervention. More specifically, massive transfusion protocols have moved away from managing a late dilutional coagulopathy. Historically in-hospital transfusion protocol managed the result of large volume crystalloid and PRBC transfusion [14]. To illustrate this, in one UK MTC, mortality reduced from 50 to 26% over a 6-year period and transfusion of blood product halved [14]. Local variation in major transfusion protocols confounds comparisons between each MTC.

Similarly, advances in pre-hospital ambulance practice, such as: technical skills around appreciation of clot preservation, pelvic binding, prioritisation of TXA administration and intra-osseous access have developed [50]. The CRASH-2 trial has shown that administration of TXA to bleeding trauma patients who are within 3 h of injury, significantly reduces all-cause mortality and death due to bleeding (risk ratio (RR) = 0.72, 95% CI 0.63, 0.83). Other potential confounders such as body temperature and pre-hospital anaesthetic agents/co-medications are not reported.

Loss to follow up, and incomplete patient records from both the pre-hospital and in-hospital phases, produced substantial missing data. Notably, 26% of follow up data is missing in the crystalloid group. To address this, MI of 10 datasets was employed [39, 44, 51]. However, it is likely that the incidence of Code Red patients in the region is slightly underestimated; due to incident proximity some patients will be transferred directly to an MTC by land ambulance, without HEMS input. In addition, if the transit time was short, patients seen by HEMS may trigger a massive transfusion on arrival at hospital, with no time to perform pre-hospital transfusion, therefore effectively removing the patient from the inclusion criteria used in this study. This study would be strengthened if the approximate point of injury (999 time) had been recorded in relation to the transfusion of PRBC, and total pre-hospital time, as opposed to the ‘on scene’ surrogate given.

A case can be argued for following the intensive care principle of ‘critical care without walls’; treating the Code Red patient on the basis of clinical need and not geographical location [52]. Future comparison studies are likely complicated by the administration of different types and quantity of blood product across services (e.g. Fibrinogen, FFP, FDP), however, collaborative prospective research amongst UK HEMS will provide larger sample sizes and generate further discussion. It may be more important that future work targets precision resuscitation in the coagulopathic patient. Improved diagnostics and therapeutics at the scene as adjuncts to current strategies are warranted, enabling focused delivery of blood products at the point of injury.

## Conclusion

In a single centre, retrospective UK HEMS study, observed mortality at 6 h and 28 days is reduced in a group of patients with suspected traumatic haemorrhage who received a PRBC transfusion compared to crystalloid. This is also reflected in a massive transfusion subgroup; however, both are statistically non-significant. Patients receiving pre-hospital PRBC were significantly less likely to need an in-hospital major transfusion compared to those receiving pre-hospital crystalloid. Further multi-centre prospective research, with adequate power to detect a true difference in patient survival, is required to establish the optimum strategy for pre-hospital volume replacement in patients with traumatic haemorrhage.

## Abbreviations

ATC: Acute Trauma Coagulopathy
CI: Confidence Interval
FDP: Freeze dried plasma
FFP: Fresh frozen plasma
GCS: Glasgow Coma Score
HEMS: Helicopter Emergency Medical Services
IQR: Interquartile Range
ISS: Injury Severity Score
KSSAAT: Kent, Surrey & Sussex Air Ambulance Trust
MI: Multiple Imputation
MODS: Multiple organ dysfunction
MOI: Mechanism of injury
MTC: Major Trauma Centre
NIHR: National Institute for Health Research
OR: Odds Ratio
PRBC: Packed red blood cells
ROSC: Return of spontaneous circulation
ROTEM: Rotational Thromboelastometry
SBP: Systolic blood pressure
SD: Standard deviation
TARN: Trauma Audit Research Network
TEG: Thromboelastography
TIC: Trauma Induced Coagulopathy
